# Lifestyle changes and risk of tuberculosis in patients with type 2 diabetes mellitus: A nationwide cohort study

**DOI:** 10.3389/fendo.2022.1009493

**Published:** 2022-10-19

**Authors:** Jiho Park, Ji Hyun Yoon, Hyun Kyun Ki, Kyungdo Han, Hyungjin Kim

**Affiliations:** ^1^ Department of Medicine, Konkuk University of Medical Center, School of Medicine, Konkuk University, Seoul, South Korea; ^2^ Department of Statistics and Actuarial Science, Soongsil University, Seoul, South Korea; ^3^ Department of Medical Humanities, Samsung Medical Center, School of Medicine, Sungkyunkwan University, Seoul, South Korea

**Keywords:** diabetes mellitus, smoking, alcohol intake, exercise, tuberculosis

## Abstract

We investigated the impacts of lifestyle changes, namely, smoking, alcohol intake, and exercise, on the development of tuberculosis (TB) in patients with type 2 diabetes mellitus (T2DM). A retrospective population-based cohort study used data from the Korean National Health Insurance system database. We examined subjects diagnosed with T2DM and without previous history of TB between 2009 and 2012 who underwent two serial health examinations. The study participants were classified into each of the four groups based on changes in the patterns of smoking, alcohol intake, and exercise at the time of the second examination. The outcome of the study was newly diagnosed TB in patients with T2DM. Among 1,659,804 included subjects, TB was newly diagnosed with 10,288 subjects. Both consistent smokers (HR 1.406; 95% CI 1.333–1.483) and new smokers (HR 1.185; 95% CI 1.063–1.320) had a higher TB risk than smoking quitters (HR 1.107; 95% CI 1.009–1.216) and never smokers. Both consistent heavy drinkers (HR 1.281; 95% CI 1.172–1.399) and heavy drinking quitters (HR 1.247; 95% CI 1.147–1.356) had a higher TB risk than new heavy drinkers and never drinkers. With respect to exercise, persistent non-exercisers (HR 1.309; 95% CI 1.72–1.399) and exercise quitters (HR 1.164; 95% CI 1.066–1.271) had a higher TB risk than new exercisers. In the subgroup analysis, a significant interaction was observed between lifestyle changes and age. We found that lifestyle changes were associated with development of TB in patients with T2DM. These results suggest that lifestyle management could be a valuable strategy for control of TB in Korea.

## Introduction

Tuberculosis (TB), the leading cause of death globally from an infectious disease, continues to be a major public health problem (1). Type 2 diabetes mellitus (T2DM) is a highly prevalent disease worldwide as a result of aging, rapid urbanization, changes in diet, and sedentary lifestyle patterns in parallel with increasing obesity (2). The global increase in T2DM is regarded as one of the emerging risk factors of controlling TB (3). Patients with T2DM are at a high risk of developing TB, particularly in countries with a high burden of both diseases in several studies (4–13). The Republic of Korea (hereafter referred to as “Korea”) has a very high prevalence of TB and T2DM. It has the highest TB incidence rate (49 per 100,000 population) among the 38 member countries of the Organization for Economic Cooperation and Development (OECD) (14) and showed a 28% prevalence rate for T2DM in adults aged >65 years (15). Although vigorous TB control measures have reduced the incidence and death rates of TB yearly in Korea, increasing populations with T2DM have the potential to considerably challenge future TB control efforts. Though there are many drugs that showed good efficacy in hyperglycemic control, lifestyle changes are still regarded as a key element in the care of T2DM patients. Smoking, alcohol intake, and regular exercise are considered essential components of lifestyle changes (16, 17). However, there were few studies on whether lifestyle changes to a desirable direction are related to developing TB in patients with T2DM. In this study, we aimed to investigate the association between lifestyle changes in patients with T2DM and development of TB using data from the National Health Insurance Service (NHIS) in Korea.

## Methods

### Source of data and study populations

**Figure 1:**
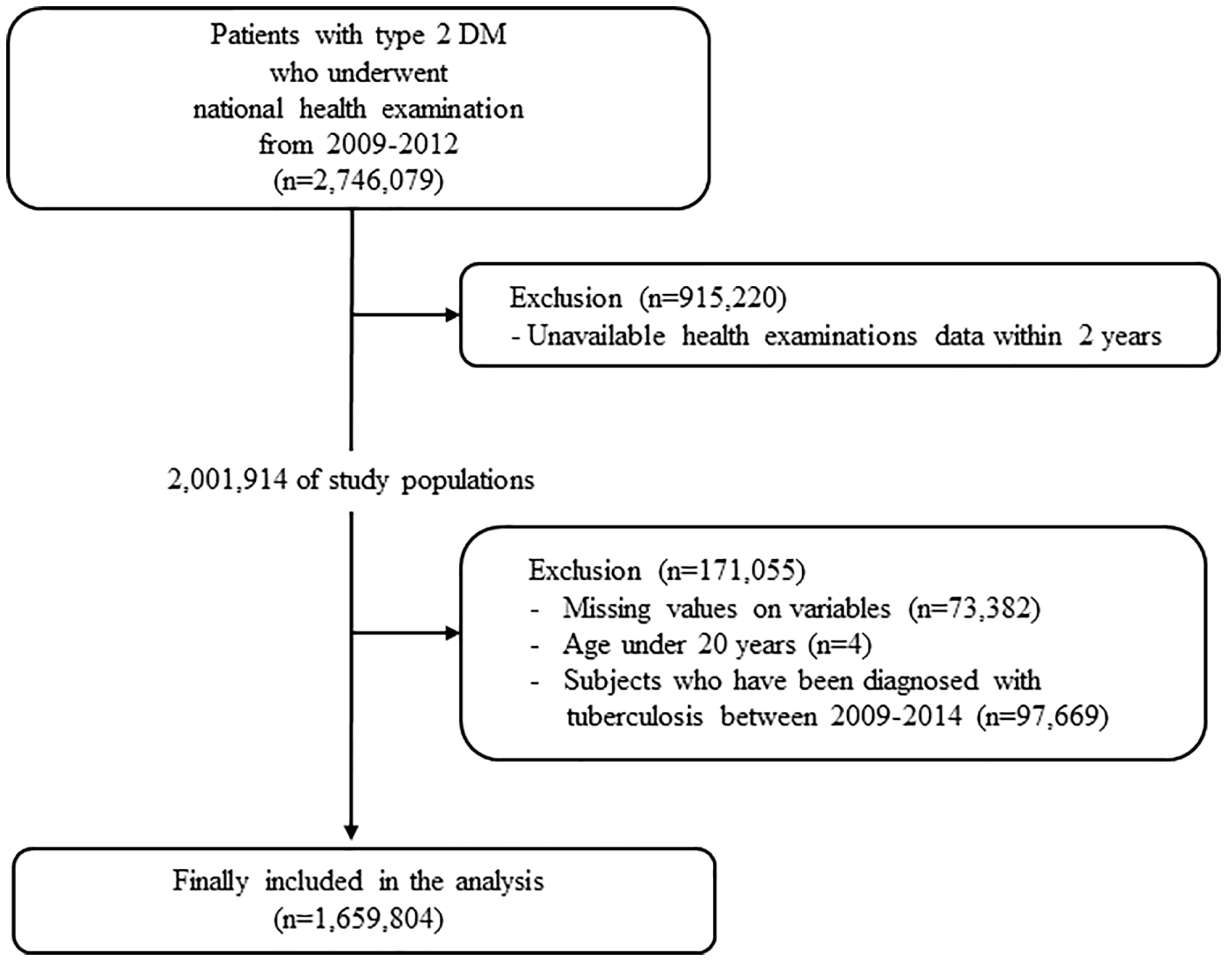
Selection of study population.

This study used data obtained from the Korean NHIS and claims database. In Korea, NHIS, a single insurer managed by the Korean government, provides a mandatory universal health insurance to 97% of the population. The NHIS provides free biennial national health examination programs for the public, and adults are recommended to undergo a check-up at least once every 2 years. For this study, we used the general health examination data and NHIS claims data including diagnosis and prescription records. This study was conducted according to the Declaration of Helsinki and was approved by the Institutional Review Board of Konkuk University of Medical Center (#2022-07-065). The IRB has approved a waiver of the requirement to obtain informed consent because the NHIS database is available to researchers, and supplied anonymized data. Patients with T2DM were defined as follows (1): patients with claims for the International Classification of Disease-10th revision (ICD-10) codes for type 2 DM (E11–E14) with at least one prescription of hypoglycemic agents or insulin or (2) patients who underwent a fasting glucose level test (≥126 mg/dl) during a health examination and were newly diagnosed with T2DM (ICD-10 code E11–E14). We screened 2,746,079 T2DM patients who underwent national health examination between 2009 and 2012. Subjects who did not undergo a follow-up health examination within 2 years after their baseline examination were subsequently excluded (*n* = 915,220). We excluded 97,669 subjects with a diagnosis of TB from the first and second health examinations. Furthermore, subjects with any missing data or age under 20 years were excluded (*n* = 73,386). Finally, 1,659,804 subjects were ultimately included in the analysis and followed up to 31 December 2018. The incidence of TB was analyzed using the claims data from 2012 to 2018 (**Figure 1**).

### Definitions of lifestyles and other covariates

Study populations responded to a standardized self-administered questionnaire provided by NHIS regarding past medical history and lifestyle behaviors such as smoking, alcohol intake, and exercise. Smoking status was classified into never smoker, ex-smoker, and current smoker. Alcohol intake was divided into none, mild to moderate (<30 g of alcohol/day), and heavy (≥30 g/day) intake. Regular exercise was defined as moderate-intensity exercise for more than 30 min or vigorous intensity exercise for more than 20 min at least once a week (18, 19). During the national health examination, height, weight, and waist circumference (WC) were measured. Body mass index (BMI) was calculated by dividing weight (kg) by height (m) squared. Fasting blood glucose, serum creatinine, and total cholesterol were measured after overnight fasting. The estimated glomerular filtration rate (eGFR) was calculated using the equation from Modification of Diet in Renal Disease Study (20). Baseline comorbidities included hypertension (HTN) and dyslipidemia. These diseases were defined using physician diagnosis or use of medication based on self-reporting. HTN was defined as systolic blood pressure (BP) ≥ 140 mmHg, diastolic BP ≥ 90 mmHg, or use of antihypertensive drugs with a prior diagnosis. Dyslipidemia was defined as a prior diagnosis (ICD-10- code E78) and treatment with statins or as total cholesterol ≥ 240 mg/dl.

### Definitions and categorization of study population according to lifestyle changes

**Figure 2:**
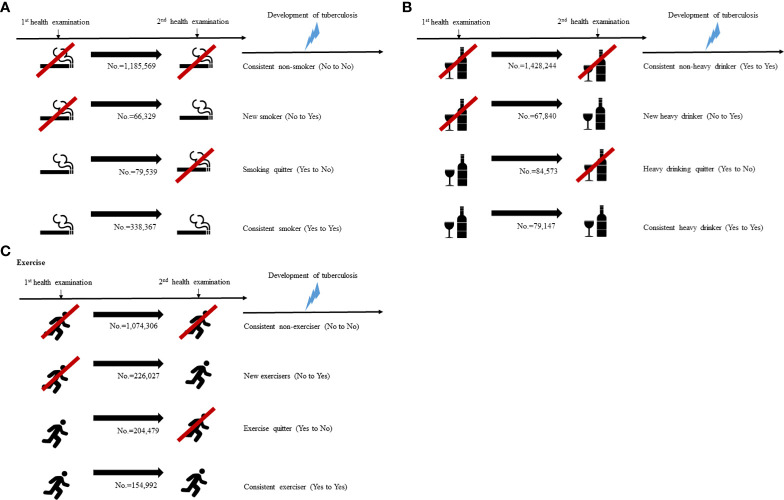
Categorization of study population according to the lifestyle changes **(A)** smoking, **(B)** alcohol intake, **(C)** exercise.

The study participants were classified into four groups based on changes of their lifestyles apparent at the time of second health examination. Four groups based on changes of smoking patterns are the following: (1) persistent non-smoker: continuously never smoking, (2) new smoker: non-smoking to current smoking, (3) smoking quitter: current smoking to non-smoking, and (4) consistent smoker or current smoker: continuously smoking. Four groups based on changes of alcohol intake patterns are the following: (1) persistent non-heavy drinker: continuously non- or mild drinking, (2) new heavy drinker: non- or mild drinking to heavy drinking, (3) heavy drinking quitter: heavy drinking to non- or mild drinking, and (4) consistent heavy drinker: continuously heavy drinking. Four groups based on changes of exercise levels are the following: (1) persistent non-exerciser: continuously non-exercising, (2) new exerciser: non-exercising to regular exercising, (3) exercise quitter: regular exercising to non-exercising, and (4) consistent exerciser: continuously regular exercising (**Figure 2**).

### Study outcomes and follow-up

The outcome of the study was occurrence of newly diagnosed TB in patients with T2DM. The NHIS provided additional insurance coverage for all patients diagnosed with TB to enhance benefit coverage from the year 2010. Specific insurance codes were applied mandatorily to patients with TB after confirmation of diagnosis (21). The cohort was followed up after the second examination date to the date of occurrence of TB or until the end of the study period.

### Statistical analysis

Anthropometric and laboratory data from the baseline examination was used in analyses. Continuous variables were presented as mean ± standard deviation and categorical variables were expressed as percentages. Clinical characteristics between the participants were compared using one-way analysis of variance for continuous variables and the chi-square test for categorical variables, respectively. The incidence rate of TB is presented per 1,000 person-years (PY). Multivariate Cox proportional-hazards regression analysis was used to estimate hazard ratios (HRs) and 95% confidence intervals (CIs) for the associations between lifestyle changes and newly diagnosed TB. The adjusted HR was calculated after adjusting the following variables: age, sex, income, HTN, dyslipidemia, BMI, GFR, fasting glucose, undergoing insulin injection, a number of anti-diabetes agents, T2DM duration, and lifestyle changes except the lifestyle patterns to be analyzed. In subgroup analysis, we stratified the participants by age (65 years and older) and by DM duration using Cox models. All reported *p*-values were two-tailed and <0.05 were considered to be statistically significant. All statistical analyses were performed using SAS version 9.3 (SAS Institute Inc., Cary, NC, USA) and R version 3.2.3 (http://www.Rproject.org; The R Foundation for Statistical Computing, Vienna, Austria).

## Results

### Baseline characteristics

Of the total 1,659,802 patients with T2DM, the mean age of the study population was 58.75 ± 11.78 years and 1,010,421 (60.88%) of the study population were male. During 9,988,951.73 PY of follow-up, a total of 10,288 of TB events occurred. **Table 1** presents the baseline characteristics of the overall study population. The baseline characteristics of the study population according to each lifestyle behavior are shown in **Supplementary Tables 1**-**3**.

**Table 1:** Baseline characteristics of overall study populations.

	Total population (*n* = 1,659,804)
**Demographics**	
Sex (male)	1,010,421 (60.8)
Age	58.75 ± 11.78
Low-income level^a^	271,182 (16.34)
**Medical history**	
Hypertension	959,506 (57.81)
Dyslipidemia	758,169 (45.68)
Pharmacologic therapy for diabetes	
Insulin	190,063 (11.45)
A number of anti-diabetes agents	
0	573,310 (34.54)
1	266,866 (16.08)
2	454,046 (27.36)
3	365,582 (22.03)
Duration of diabetes (years)	4.16 ± 3.95
**Physical exam**	
BMI (kg/m^2^)	24.95 ± 3.3
BP (mmHg)	127.96 ± 15.15
DBP (mmHg)	78.15 ± 9.91
**Laboratory findings**	
Fasting glucose (mg/dL)	133.23 ± 45.53
Total cholesterol (mg/dL)	189.56 ± 43.05
GFR (ml/min/1.73 m^2^)	87.1 ± 40.98
**Lifestyles**	
Smoking	
Non	922,120 (55.56)
Ex	342,988 (20.66)
Current	394,696 (23.78)
Alcohol intake	
Non	950,927 (57.29)
Mild	561,890 (33.85)
Heavy	146,987 (8.86)
Regular exercise	381,019 (22.96)

Values are presented as mean ± standard deviation and number (%).

BMI, body mass index; SBP, systolic blood pressure; DBP, diastolic blood pressure; GFR, glomerular filtration rates.

a Low 20% and recipient of medical aid.

### Risk for development of tuberculosis according to the lifestyle changes during follow-up

We investigated the relationship between the lifestyle changes and the development of TB during follow-up. The associations between lifestyle changes and development of TB are demonstrated in **Table 2**. With respect to smoking, the incidence rates (IRs) of TB in each group were 0.98635, 1.06268, 1.04371, and 1.1773 per 1,000 PY, respectively. As expected, consistent smoker showed the highest IRs of TB and showed the same result after multivariate analysis (HR 1.406; 95% CI 1.333–1.483). As compared with new smokers (HR 1.185; 95% CI 1.063–1.320), smoking quitters recently had a lower TB risk (HR 1.107; 95% CI 1.009–1.216). With respect to alcohol intake, the IRs of TB in each group were 1.00931, 1.05456, 1.22404, and 1.17788 per 1,000 PY, respectively. As compared with heavy drinking quitters (HR 1.247, 95% CI 1.147–1.356), new heavy drinkers had a lower TB risk (HR 1.122; 95% CI 1.016–1.239). With respect to exercise, the IRs of TB in each group were 1.07068, 0.95759, 1.02286, and 0.86752 per 1,000 PY, respectively. Consistent exercisers showed the lowest IRs of TB as expected. Persistent non-exercisers during the follow-up period showed the highest IRs of TB and showed same result after multivariate analysis (HR 1.309; 95% CI 1.216–1.408). As compared with exercise quitters (HR 1.164, 95% CI 1.066–1.271), new exercisers had a lower TB risk (HR 1.159; 95% CI 1.062–1.265).

**Table 2:** The associations between lifestyle changes and development of TB.

	No.	TB	Follow-up duration,person-years	IR per 1000	Model 1^a^, HR(95% CI)	Model 2^b^, HR(95% CI)	Model 3^c^, HR(95% CI)
**Smoking**							
Consistent non-smoker	1,185,569	7,075	7,172,908	0.98635	1 (ref.)	1 (ref.)	1 (ref.)
New smoker	56,329	360	338,767.47	1.06268	1.079 (0.97–1.199)	1.189 (1.067–1.324)	1.185 (1.063–1.32)
Smoking quitter	79,539	496	475,229.3	1.04371	1.06 (0.968–1.161)	1.118 (1.018–1.227)	1.107 (1.009–1.216)
Consistent smoker	338,367	2,357	2,002,046.96	1.1773	1.199 (1.144–1.256)	1.425 (1.352–1.502)	1.406 (1.333–1.483)
**Alcohol intake**							
Consistent non-heavy drinker	1,428,244	8,688	8,607,840.84	1.00931	1 (ref.)	1 (ref.)	1 (ref.)
New heavy drinkers	67,840	428	405,855.52	1.05456	1.047 (0.95–1.153)	1.17 (1.06–1.291)	1.122 (1.016–1.239)
Heavy drinking quitter	84,573	617	504,069.25	1.22404	1.215 (1.12–1.319)	1.287 (1.184–1.4)	1.247 (1.147–1.356)
Consistent heavy drinker	79,147	555	471,186.13	1.17788	1.17 (1.074–1.275)	1.348 (1.234–1.472)	1.281 (1.172–1.399)
**Exercise**							
Consistent non-exerciser	1,074,306	6,883	6,428,651.26	1.07068	1.238 (1.152–1.331)	1.359 (1.264–1.462)	1.309 (1.216–1.408)
New exerciser	226,027	1,313	1,371,156.87	0.95759	1.105 (1.013–1.206)	1.179 (1.08–1.286)	1.159 (1.062–1.265)
Exercise quitter	204,479	1,270	1,241,616.32	1.02286	1.18 (1.081–1.288)	1.187 (1.087–1.296)	1.164 (1.066–1.271)
Consistent exerciser	154,992	822	947,527.29	0.86752	1 (ref.)	1 (ref.)	1 (ref.)

TB, tuberculosis; IR: incidence rate; HR: hazard ratio; CI: confidence interval; BMI: body mass index.

a Model 1: Non-adjusted.

b Model 2: adjusted for age, sex, income, hypertension, dyslipidemia, fasting glucose, glomerular filtration rates, a number of anti-diabetes agents, diabetes duration.

c Model 3: Model 2 + lifestyle changes except the lifestyle patterns to be analyzed.

### Subgroup analysis according to age and DM duration

**Figure 3:**
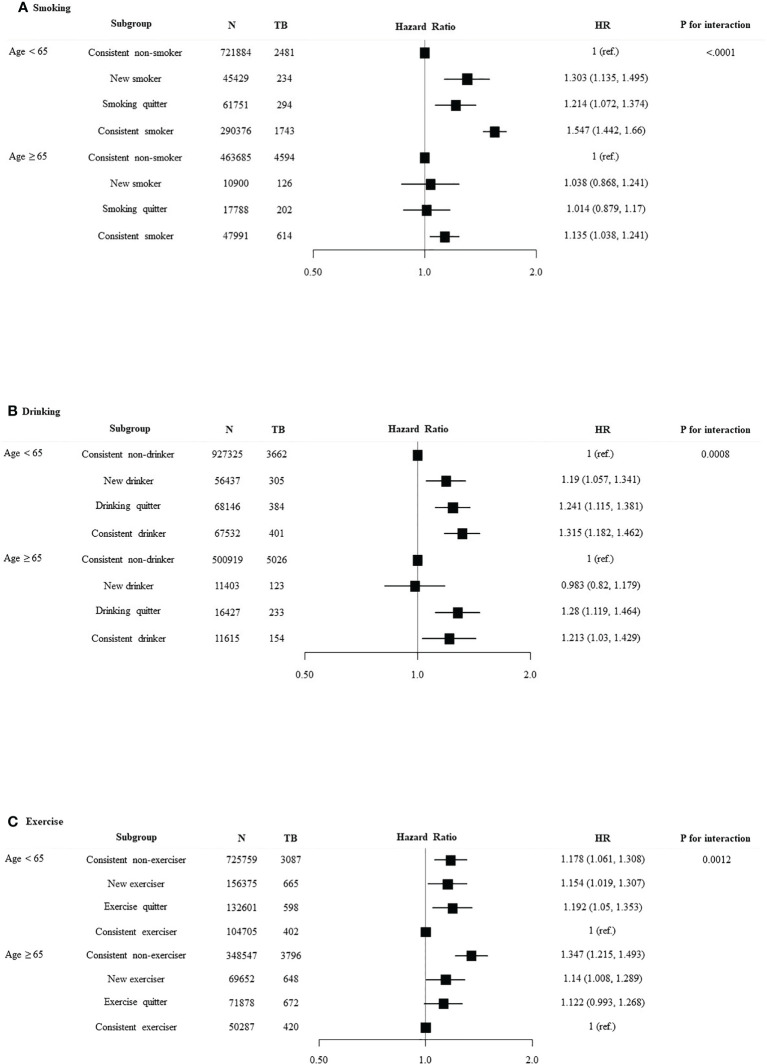
Relationship between lifestyle changes and development of TB according to age. Subgroup analyses were performed using Cox proportional-hazards regression analysis. **(A)** Smoking, **(B)** alcohol intake, and **(C)** exercise.

**Figure 4:**
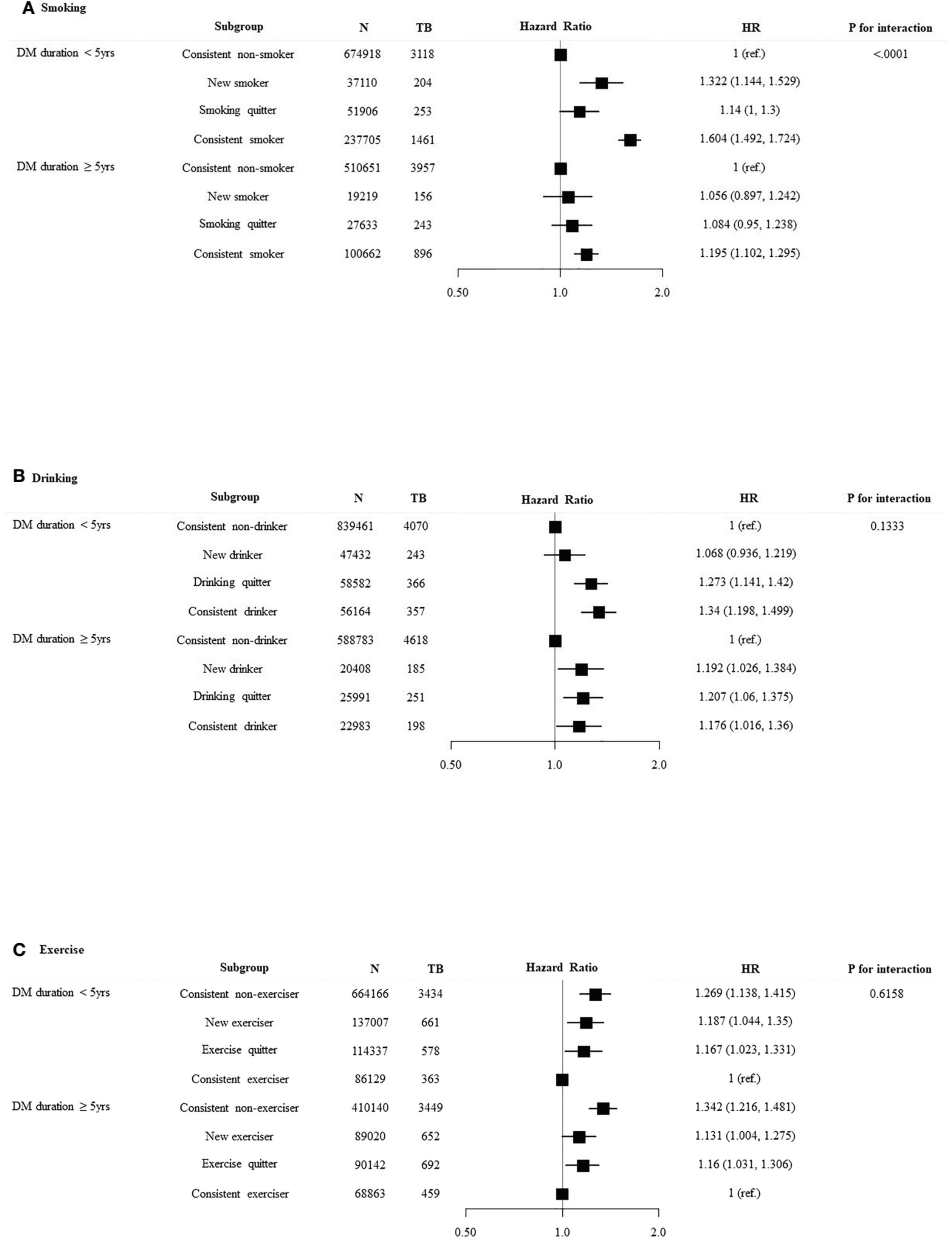
Relationship between lifestyle changes and development of TB according to DM duration. Subgroup analyses were performed using Cox proportional-hazards regression analysis. **(A)** Smoking, **(B)** alcohol intake, and **(C)** exercise.

We divided the patients into age < 65 years and age ≥ 65 years and investigated the association between lifestyle changes and development of TB (**Figure 3**). Significant association was observed between age and smoking (*p* for interaction < 0.0001). In age < 65 years group, new smokers (HR 1.303, 95% CI 1.135–1.495) and smoking maintainers (HR 1.547, 95% CI 1.442–1.66) had an increased risk of TB. Significant interaction was identified between age and alcohol intake (*p* for interaction = 0.0008). With respect to the age < 65 years group, consistent heavy drinkers (HR 1.315, 95% CI 1.182–1.462) demonstrated a substantially higher TB risk than persistent non-heavy drinkers. There was a significant interaction between age and the risk of TB according to exercise pattern (*p* for interaction = 0.0012). Conversely, in the age ≥ 65 years group, exercises quitters (HR 1.122, 95% CI 0.993–1.268) and persistent non-exercisers (HR 1.347, 95% CI 1.215–1.493) had an increased risk of TB. We divided the patients into DM duration < 5 years and DM duration ≥ 5 years and investigated the association between lifestyle changes and development of TB (**Figure 4**). Significant association was observed between T2DM duration and smoking (*p* for interaction < 0.0001). With respect to patients with DM duration <5 years, new smokers (HR 1.322, 95% CI 1.144–1.529) and consistent smokers (HR 1.604, 95% CI 1.492–1.724) demonstrated a substantially higher TB risk than persistent non-smokers and smoking quitters.

## Discussion

In this nationwide population-based cohort study, we comprehensively investigated the association between lifestyle changes and newly diagnosed TB in patients with T2DM. The main findings of our study are as follows: (1) consistent smokers and new smokers have a higher risk of TB than persistent non-smokers, (2) consistent heavy drinkers and heavy drinking quitters have a higher risk of TB than persistent non-drinkers, (3) persistent non-exercisers and exercise quitters have a higher risk of TB than consistent exercisers, and (4) in the subgroup analysis, a significant interaction was observed between lifestyle changes and age.

Smoking was identified as an independent risk factor of TB in populations with DM and without DM according to previous studies (9, 22–24). Smoking years was regarded as a risk factor for TB in a positive dose response manner among ever-smokers (25). With a slightly conflicting concept, smoking cessation was also helpful to reduce the risk of TB mortality to a level not different from never smokers (23, 26). Although there were no data on how many cigarettes smoked in the past by ex-smokers, given the large number of study participants, our findings suggest that that the new smokers, irrespective of the amount of cigarettes, might harbor a higher risk of developing TB than the smoking quitters, although the 95% CIs of the two groups still overlap. This association between smoking patterns and TB was more significant in those under 65 years of age and DM duration <5 years. It could be seen that younger people or patients with DM duration <5 years have the benefit of reducing the development of TB by quitting smoking. This suggests that old age itself and longer DM duration are not only risk factors for TB, but that they are less resilient to changes in the immune system caused by lifestyle changes such as smoking (27). It has been assumed that approximately 10% of TB cases are probably due to heavy alcohol intake with or without DM. It is still not known whether the increased risk of TB is attributable to the alcohol itself or because of the sequelae of alcohol abuse, such as liver damage and nutritional deficiency, or socio-economic factors. Although there have been debatable findings related to various confounding factors, the association between alcohol intake and TB has been found. Several studies carried out *in vitro* as well as *in vivo* have demonstrated that excessive alcohol intake significantly alters the immune system, vulnerable to respiratory diseases such as TB (22, 28–30). Contrary to smoking, heavy drinking quitters have a higher risk of developing TB than new drinkers. It could be explained by the sick quitter effect, which is attributed to quitting or reducing drinking due to health problems, which could be risk factors for TB. Several studies have reported an association between health status and alcohol intake. Hospitalization, chronic conditions such as alcohol-induced liver injury, and major medical diagnosis were associated with fewer drinks per month (31). Another interesting finding in this study was that persistent non-exercisers and exercise quitters have a higher risk of developing TB than new exercisers and consistent exercisers. Physical inactivity was known to increase the risk of major diseases such as cardiovascular disease, T2DM, or cancers (32). Although there was scarce evidence of association between physical inactivity and infectious disease including TB, it seems likely that exercise can affect TB risk in terms of immune function (33). A few studies showed that patients with or without T2DM who continue to exercise had a relatively low risk of TB (34, 35). Other studies showed that the risk of TB among the elderly increased with physical inactivity (18). Our study provided informative data that the changes of exercise patterns in the direction of inactivity increased the risk of TB in T2DM patients regardless of glycemic control. The prevalence of T2DM worldwide has risen about 20% in the past three decades, particularly in newly industrialized countries that are undergoing a rapid economic and demographic transition, including Korea. Several epidemiological studies have concluded that T2DM increases the risk of TB by about threefold, mainly by impairing the host-defense mechanism and thereby increasing the risk of development from latent TB infection to active infection (10, 36, 37). TB is a preventable and curable disease; therefore, efforts for control of TB as a global health concern need to be expanded and intensified (37). Every community also needs to assess the prevalence of T2DM and its attribution to TB at the population level (37, 38). Among the known risk factors for TB in T2DM patients, a new strategy for control TB that can rectify correctable factors is needed, and lifestyle changes suggested in our study would be representative correctable factors among them.

Several limitations are evident in this study. First, we determined the changes of lifestyles of the study population during the first two healthy examinations. There is insufficient evidence as to whether the lifestyle determined through two consecutive health examinations every 2 years was consistent during the follow-up period. Second, the self-reported nature of the health examination questionnaire asking about private lifestyles would be affected by recall bias. Third, there is a lack of comprehensive analysis between lifestyle changes on TB occurrence. In fact, it could be ambiguous to view each lifestyle change as an absolute independent risk factor for tuberculosis occurrence. There may be complex interactions in the human body between lifestyle habits, diabetes, and tuberculosis, which cannot be found statistically or through disease research. Fourth, unavailable confounders might not be fully adjusted for. Fifth, as this study included only Korean populations where the incidence of TB is high and metabolic characteristics are related to T2DM, it might be different from other ethnicities. Therefore, the study results might not be generalizable to other populations from other countries. Sixth, we did not include type 1 DM patients in this study; thus, the results could not be applicable to patients with type 1 DM. Nevertheless, the relatively large sample size of our study and the utilization of administrative data using standardized protocols could be considered strengths of this study. Until now, no study has evaluated the relationship between changes in the pattern of smoking, alcohol intake, and regular exercise in patients with T2DM *via* a longitudinal design. Study results are essential to guide researchers as regards the most efficient use of limited resources for TB control.

## Conclusions

In this large nationwide cohort study, we found that lifestyle changes were associated with the development of TB in patients with T2DM. These results suggest that lifestyle management could be a valuable strategy for control of TB in Korea.

## Data availability statement

The original contributions presented in the study are included in the article/**Supplementary Material**. Further inquiries can be directed to the corresponding authors.

## Ethics statement

The studies involving human participants were reviewed and approved by Konkuk University of Medical Center. Written informed consent for participation was not required for this study in accordance with the national legislation and the institutional requirements.

## Author contributions

JP, HJK, and KH. conceived the presented idea. KH collected the study data and did the statistical analyses. JP wrote the initial draft of the paper. JP, JY, HKK, and HJK reviewed the manuscript. HJK and KH supervised the manuscript and acted as senior authors. All authors approved the paper.

## Conflict of interest

The authors declare that the research was conducted in the absence of any commercial or financial relationships that could be construed as a potential conflict of interest.

## Publisher’s note

All claims expressed in this article are solely those of the authors and do not necessarily represent those of their affiliated organizations, or those of the publisher, the editors and the reviewers. Any product that may be evaluated in this article, or claim that may be made by its manufacturer, is not guaranteed or endorsed by the publisher.
