# Supplementary material for: Lifestyle changes and risk of tuberculosis in patients with type 2 diabetes mellitus: A nationwide cohort study
Source: Front Endocrinol (Lausanne). 2022 Oct 19;13:1009493. doi: 10.3389/fendo.2022.1009493 (PMC9627208; doi:10.3389/fendo.2022.1009493)
Supplement: Supplementary file 1 [file Table_1.docx]

**Supplementary Table 1. Baseline characteristics of the overall participants based on smoking patterns**

|  | **Smoking** | | | |
| --- | --- | --- | --- | --- |
|  | **consistent**  **non-smoker** | **new smoker** | **smoking quitter** | **consistent smoker** |
|  | **(n=1185569)** | **(n=56329)** | **(n=79539)** | **(n=338367)** |
| **Demographics** |  |  |  |  |
| Sex (male) | 562,524 (47.45) | 51170 (90.84) | 72264 (90.85) | 324463 (95.89) |
| Age | 60.96 ± 11.23 | 54.69 ± 11.2 | 55.69 ± 11.42 | 52.4 ± 11.19 |
| Low-income level | 195992 (16.53) | 9034 (16.04) | 12400 (15.59) | 53756 (15.89) |
| **Medical history** |  |  |  |  |
| Hypertension | 725972 (61.23) | 29043 (51.56) | 42606 (53.57) | 161885 (47.84) |
| Dyslipidemia | 567972 (47.91) | 23125 (41.05) | 34965 (43.96) | 132107 (39.04) |
| **Pharmacologic therapy for diabetes** | | | | |
| Insulin | 145900 (12.31) | 5418 (9.62) | 10815 (13.6) | 27930 (8.25) |
| A number of anti-diabetes agents | | | | |
| 0 | 361058 (30.45) | 22569 (40.07) | 30844 (38.78) | 158839 (46.94) |
| 1 | 211013 (17.8) | 7328 (13.01) | 10463 (13.15) | 38062 (11.25) |
| 2 | 341712 (28.82) | 14341 (25.46) | 20260 (25.47) | 77733 (22.97) |
| 3 | 271786 (22.92) | 12091 (21.46) | 17972 (22.6) | 63733 (18.84) |
| Duration of diabetes | 4.5 ± 3.97 | 3.62 ± 3.8 | 3.66 ± 3.85 | 3.16 ± 3.75 |
| **Physical exam** |  |  |  |  |
| BMI | 25.01 ± 3.29 | 24.83 ± 3.23 | 24.97 ± 3.21 | 24.78 ± 3.34 |
| SBP | 128.4 ± 15.31 | 126.52 ± 14.71 | 127.25 ± 14.74 | 126.83 ± 14.69 |
| DBP | 77.91 ± 9.87 | 78.34 ± 10.01 | 78.53 ± 9.96 | 78.89 ± 9.99 |
| **Laboratory findings** |  |  |  |  |
| Fasting glucose | 131.62 ± 42.77 | 136.06 ± 49.92 | 137.08 ± 50.29 | 137.51 ± 52.12 |
| Total cholesterol | 188.51 ± 42.32 | 189.89 ± 43.43 | 190.26 ± 47.77 | 193.02 ± 44.15 |
| GFR | 85.36 ± 38.18 | 89.87 ± 42.22 | 88.93 ± 47.17 | 92.3 ± 47.65 |
| **Lifestyles** |  |  |  |  |
| Smoking |  |  |  |  |
| Non | 895751 (75.55) | 0 (0) | 26369 (33.15) | 0 (0) |
| Ex | 289818 (24.45) | 0 (0) | 53170 (66.85) | 0 (0) |
| Current | 0 (0) | 56329 (100) | 0 (0) | 338367 (100) |
| Alcohol intake |  |  |  |  |
| Non | 805607 (67.95) | 17659 (31.35) | 35789 (45) | 91872 (27.15) |
| Mild | 316129 (26.66) | 29734 (52.79) | 34393 (43.24) | 181634 (53.68) |
| Heavy | 63833 (5.38) | 8936 (15.86) | 9357 (11.76) | 64861 (19.17) |
| Regular exercise | 282262 (23.81) | 12744 (22.62) | 18929 (23.8) | 67084 (19.83) |

Abbreviations : BMI, body mass index; SBP, systolic blood pressure; DBP, diastolic blood pressure; GFR, glomerular filtration rates
