# Supplementary material for: Lifestyle changes and risk of tuberculosis in patients with type 2 diabetes mellitus: A nationwide cohort study
Source: Front Endocrinol (Lausanne). 2022 Oct 19;13:1009493. doi: 10.3389/fendo.2022.1009493 (PMC9627208; doi:10.3389/fendo.2022.1009493)
Supplement: Supplementary file 2 [file Table_2.docx]

**Supplementary Table 2. Baseline characteristics of the overall participants based on alcohol intake patterns**

|  | **Alcohol intake** | | | |  |
| --- | --- | --- | --- | --- | --- |
|  | **consistent**  **non-heavy drinker** | **new**  **heavy drinker** | **heavy drinking quitter** | **consistent**  **heavy drinker** |  |
|  | **(n=1428244)** | **(n=67840)** | **(n=84573)** | **(n=79147)** |  |
| **Demographics** |  |  |  |  |  |
| Sex (male) | 786057 (55.04) | 65236 (96.16) | 81152 (95.95) | 77976 (98.52) |  |
| Age | 59.47 ± 11.77 | 54.08 ± 10.88 | 54.93 ± 11.09 | 53.74 ± 10.4 |  |
| Low-income level | 238359 (16.69) | 9761 (14.39) | 12392 (14.65) | 10670 (13.48) |  |
| **Medical history** |  |  |  |  |  |
| Hypertension | 824702 (57.74) | 39090 (57.62) | 48699 (57.58) | 47015 (59.4) |  |
| Dyslipidemia | 666205 (46.65) | 26588 (39.19) | 33919 (40.11) | 31457 (39.75) |  |
| **Pharmacologic therapy for diabetes** | | | | |  |
| Insulin | 172234 (12.06) | 4614 (6.8) | 8213 (9.71) | 5002 (6.32) |  |
| A number of anti-diabetes agents | | | | | |
| 0 | 466547 (32.67) | 31612 (46.6) | 37506 (44.35) | 37645 (47.56) |  |
| 1 | 236843 (16.58) | 8908 (13.13) | 11024 (13.03) | 10091 (12.75) |  |
| 2 | 399778 (27.99) | 15913 (23.46) | 20098 (23.76) | 18257 (23.07) |  |
| 3 | 325076 (22.76) | 11407 (16.81) | 15945 (18.85) | 13154 (16.62) |  |
| Duration of diabetes | 4.32 ± 3.96 | 3.2 ± 3.72 | 3.25 ± 3.75 | 3.08 ± 3.69 |  |
| **Physical exam** |  |  |  |  |  |
| BMI | 24.91 ± 3.31 | 25.18 ± 3.19 | 25.13 ± 3.21 | 25.3 ± 3.21 |  |
| SBP | 127.64 ± 15.17 | 130.19 ± 14.98 | 129.11 ± 14.84 | 130.71 ± 14.79 |  |
| DBP | 77.75 ± 9.83 | 80.82 ± 10.03 | 79.94 ± 9.98 | 81.24 ± 10.01 |  |
| **Laboratory findings** |  |  |  |  |  |
| Fasting glucose | 132.23 ± 44.91 | 140.37 ± 49.76 | 137.12 ± 47.79 | 141.14 ± 48.71 |  |
| Total cholesterol | 188.93 ± 42.61 | 194.45 ± 46.07 | 191.13 ± 42.12 | 195.06 ± 48.23 |  |
| GFR | 86.23 ± 40.32 | 92.41 ± 46.05 | 91.61 ± 44.02 | 93.31 ± 43.56 |  |
| **Lifestyle** |  |  |  |  |  |
| Smoking |  |  |  |  |  |
| Non | 873104 (61.13) | 13732 (20.24) | 22179 (26.22) | 13105 (16.56) |  |
| Ex | 271400 (19) | 20878 (30.78) | 25235 (29.84) | 25475 (32.19) |  |
| Current | 283740 (19.87) | 33230 (48.98) | 37159 (43.94) | 40567 (51.26) |  |
| Alcohol intake |  |  |  |  |  |
| Non | 934861 (65.46) | 0 (0) | 16066 (19) | 0 (0) |  |
| Mild | 493383 (34.54) | 0 (0) | 68507 (81) | 0 (0) |  |
| Heavy | 0 (0) | 67840 (100) | 0 (0) | 79147 (100) |  |
| Regular exercise | 326519 (22.86) | 16334 (24.08) | 19933 (23.57) | 18233 (23.04) |  |

Abbreviations : BMI, body mass index; SBP, systolic blood pressure; DBP, diastolic blood pressure; GFR, glomerular filtration rates
