# Supplementary material for: Lifestyle changes and risk of tuberculosis in patients with type 2 diabetes mellitus: A nationwide cohort study
Source: Front Endocrinol (Lausanne). 2022 Oct 19;13:1009493. doi: 10.3389/fendo.2022.1009493 (PMC9627208; doi:10.3389/fendo.2022.1009493)
Supplement: Supplementary file 3 [file Table_3.docx]

**Supplementary Table 3. Baseline characteristics of the overall participants based on exercise patterns**

|  | **Exercise** | | | |  |
| --- | --- | --- | --- | --- | --- |
|  | **consistent**  **non-exerciser** | **new exerciser** | **exercise quitter** | **consistent exerciser** |  |
|  | **(n=1074306)** | **(n=226027)** | **(n=204479)** | **(n=154992)** |  |
| **Demographics** |  |  |  |  |  |
| Sex (male) | 623732 (58.06) | 146081(64.63) | 128266 (62.73) | 112342 (72.48) |  |
| Age | 58.5 ± 12.24 | 58.53 ± 11.11 | 59.86 ± 10.99 | 59.35 ± 10.34 |  |
| Low-income level | 179713 (16.73) | 36227 (16.03) | 33481 (16.37) | 21761 (14.04) |  |
| **Medical history** |  |  |  |  |  |
| Hypertension | 617768 (57.5) | 128852 (57.01) | 122267 (59.79) | 90619 (58.47) |  |
| Dyslipidemia | 492100 (45.81) | 101945 (45.1) | 95020 (46.47) | 69104 (44.59) |  |
| **Pharmacologic therapy for diabetes** | | | | | |
| Insulin | 123809 (11.52) | 25398 (11.24) | 24873 (12.16) | 15983 (10.31) |  |
| A number of anti-diabetes agents | | | | | |
| 0 | 383955 (35.74) | 76290 (33.75) | 62775 (30.7) | 50290 (32.45) |  |
| 1 | 166390 (15.49) | 38159 (16.88) | 34603 (16.92) | 27714 (17.88) |  |
| 2 | 287162 (26.73) | 62773 (27.77) | 59269 (28.99) | 44842 (28.93) |  |
| 3 | 236799 (22.04) | 48805 (21.59) | 47832 (23.39) | 32146 (20.74) |  |
| Duration of diabetes | 4.02 ± 3.93 | 4.15 ± 3.94 | 4.57 ± 3.97 | 4.57 ± 4.02 |  |
| **Physical exam** |  |  |  |  |  |
| BMI | 25.02 ± 3.39 | 24.86 ± 3.15 | 24.87 ± 3.19 | 24.74 ± 2.98 |  |
| SBP | 127.97 ± 15.28 | 127.7 ± 14.9 | 128.16 ± 15.11 | 128.08 ± 14.66 |  |
| DBP | 78.27 ± 9.98 | 77.93 ± 9.78 | 77.99 ± 9.87 | 77.89 ± 9.67 |  |
| **Laboratory findings** |  |  |  |  |  |
| Fasting glucose | 133.93 ± 47.02 | 130.82 ± 42.1 | 133.77 ± 44.78 | 131.2 ± 40.33 |  |
| Total cholesterol | 190.88 ± 43.74 | 187.61 ± 42.08 | 187.59 ± 41.91 | 185.87 ± 40.64 |  |
| GFR | 87.24 ± 40.55 | 87.27 ± 41.38 | 86.59 ± 42.11 | 86.53 ± 41.85 |  |
| **Lifestyle** |  |  |  |  |  |
| Smoking |  |  |  |  |  |
| Non | 609465 (56.73) | 120757 (53.43) | 116006 (56.73) | 75892 (48.97) |  |
| Ex | 192503 (17.92) | 55734 (24.66) | 45943 (22.47) | 48808 (31.49) |  |
| Current | 272338 (25.35) | 49536 (21.92) | 42530 (20.8) | 30292 (19.54) |  |
| Alcohol intake |  |  |  |  |  |
| Non | 629164 (58.56) | 124646 (55.15) | 119790 (58.58) | 77327 (49.89) |  |
| Mild | 349748 (32.56) | 81758 (36.17) | 67663 (33.09) | 62721 (40.47) |  |
| Heavy | 95394 (8.88) | 19623 (8.68) | 17026 (8.33) | 14944 (9.64) |  |
| Regular exercise | 0 (0) | 226027 (100) | 0 (0) | 154992 (100) |  |

Abbreviations : BMI, body mass index; SBP, systolic blood pressure; DBP, diastolic blood pressure; GFR, glomerular filtration rates
